# Development of a Novel Nordic Hamstring Exercise Device to Measure and Modify the Knee Flexors' Torque-Length Relationship

**DOI:** 10.3389/fspor.2021.629606

**Published:** 2021-02-24

**Authors:** Emma Sconce, Ben Heller, Tom Maden-Wilkinson, Nick Hamilton

**Affiliations:** ^1^Sports Engineering Research Group (SERG), Advanced Wellbeing Research Centre (AWRC), Sheffield Hallam University, Sheffield, United Kingdom; ^2^Physical Activity, Wellness and Public Health Research Group (PAWPH), Advanced Wellbeing Research Centre (AWRC), Sheffield Hallam University, Sheffield, United Kingdom

**Keywords:** Nordic hamstring exercise, hamstring strain injury, injury prevention, rehabilitation, long muscle length training

## Abstract

The Nordic hamstring exercise (NHE) has been shown to reduce hamstring injury risk when employed in training programs. This study investigates a novel device to modify the NHE torque-length relationship of the knee flexors, as targeting the hamstrings at a more extended length may have benefits for hamstring strain injury prevention and rehabilitation. Eighteen recreational male participants completed three bilateral NHE repetitions at a conventional 0° flat position, a 10° incline, and a 10° decline slope on a novel device (HA*L*HAM°). Measures of peak torque and break-torque angle explored the effect of inclination on the knee flexors' length-tension relationship. Relative thigh-to-trunk angle and angular velocity of the knee joint were used to assess influence of inclination on technique and exercise quality. Break-torque angle increased when performed at an incline (134.1 ± 8.6°) compared to both the decline (112.1 ± 8.3°, *p* <0.0001, *g* = 2.599) and standard flat NHE positions (126.0 ± 9.8°, *p* = 0.0002, *g* = 0.885). Despite this, altering inclination did not affect eccentric knee flexor peak torque (decline = 132.0 ± 63.1 Nm, flat = 149.7 ± 70.1 Nm, incline = 148.9 ± 64.9 Nm, *F* = 0.952, *p* = 0.389), angular velocity of the knee joint at break-torque angle (decline = 23.8 ± 14.4°, flat = 29.2 ± 22.6°, incline = 24.5 ± 22.6°, *F* = 0.880, *p* = 0.418) or relative thigh-to-trunk angle at break-torque angle (decline = 20.4 ± 10.4°, flat = 16.7 ± 10.8°, incline = 20.2 ± 11.2°, *F* = 1.597, *p* = 0.207). The report recommends the use of arbitrary metrics such as break-torque angle that can be replicated practically in the field by practitioners to assess proxy muscle length changes i.e., the angular range over which the torque can be produced. Inclination of the Nordic hamstring exercise leads to hamstring muscle failure at longer muscle lengths without reductions in the maximal force exuded by the muscle. Therefore, the NHE performed on an incline may be a more effective training intervention, specific to the proposed mechanism of hamstring strain injury during sprinting that occurs whilst the muscle is rapidly lengthening. Using a graded training intervention through the inclinations could aid gradual return-to-play rehabilitation.

## Introduction

Hamstring strain injuries (HSIs) are common lower limb injuries that occur in intensive, acyclic sports (Ekstrand et al., 2011) presenting an 18% re-injury rate, (Hägglund et al., 2006) worsened by poor quality formation of scar tissue in the myotendinous junction post-injury (Hägglund et al., 2006). The incidence of HSIs within men's football is still increasing annually (2.3% per year) (Ekstrand et al., 2016), despite a rise in research interest and implementation. HSIs present a major challenge in elite football, with a missed mean playing time of 14 competition days per injury resulting in substantial financial strain: and potentially having a detrimental impact on a team's performance (Ekstrand et al., 2011). More investigation is needed to explore the unexplained continuing high injury rates despite decades of inquiry into risk factors and causation. HSI occurrence is associated with the hamstrings being subject to high forces during rapid muscle lengthening actions (muscle-tendon stretch and negative work), such as in high-speed running (Opar et al., 2012). In the late swing phase, the hamstrings rapidly change from acting eccentrically (decelerating the extended knee) to concentrically (supporting hip extension). This places them in a more susceptible extended position under high mechanical stress, as the hamstrings develop maximal tension to stabilize the knee joint (Guex et al., 2012). By targeting the hamstrings in a lengthened position during eccentric hamstring training, the muscle orientation at injury can be more closely reproduced.

Studies using isometric training at longer muscle lengths have reported greater muscular hypertrophy (5–19.7%) and maximal force production (8–60.3%) when compared to equal volumes of training at shorter muscle lengths, and regardless of training intensity (Oranchuk et al., 2019). The Nordic hamstring exercise (NHE) is an eccentric-only exercise, placing load on the hamstring muscles whilst they are lengthening which has been shown to reduce HSI risk (Arnason et al., 2008; Petersen et al., 2011; Van Der Horst et al., 2015; Oranchuk et al., 2019; Van Dyk et al., 2019). However, the mechanisms that elicit these beneficial adaptations in the knee flexors are conflicting in the research and not yet fully understood (Timmins et al., 2016; Van Hooren and Bosch, 2017; Presland et al., 2018). Some researchers attribute muscle architecture changes to generation of more sarcomeres in series following eccentric bouts of training leading to a greater effective series compliance of the muscle (Brockett et al., 2001, 2004). The slow, eccentric and maximal nature of the NHE (knee flexors are overloaded past their capacity) appears to result in a true eccentric mechanism that provides a stimulus whereby the myosin heads are already attached to actin and forced to detach by the lengthening of the cross-bridges, incurring muscle damage (Franchi et al., 2017; Cuthbert et al., 2020b). The NHE action is slow enough to be regarded as quasi-static and the resisting torque can be considered as equal to the extending torque until the force-production capability of the individuals knee flexors can no longer resist the increasing torque, resulting in a “break-point” angle (the angle at which the individual can no longer resist the increasing gravitational moment and falls to the floor) (Sconce et al., 2015). An observed peak torque shift in the direction of more extended angles during long muscle-length chronic-training interventions is attributed to an increase in fascicle length, assumed to reflect more sarcomeres in series (Morgan, 1990; Potier et al., 2009; Oranchuk et al., 2019). It is proposed that in the NHE this would result in a larger break-point angle (where 180° is full extension), corresponding to a longer muscle length at failure (Brughelli and Cronin, 2007) thus potentially leading to greater increases in muscle fascicle length, and modifying the length-tension relationship of the hamstrings which may reduce injury risk.

Eccentric hamstring strength has traditionally been measured through isokinetic dynamometry testing, seen as the “gold standard” as to which to compare against, however the role of isokinetic strength assessment for detecting the risk of future HSIs is questionable (Van Dyk et al., 2017; Green et al., 2018). This may be in part because it is typically performed in a seated position within a 0–90° knee flexion to extension range. This hip position has low ecological validity and does not permit the hamstrings to be placed in a position which is representative of how injury occurs in sprinting-related activities. Isokinetic testing is also expensive and relatively inaccessible outside of research laboratories. Portable NHE devices have presented a lower cost alternative to isokinetics, offering ongoing monitoring and feedback in an applied setting (Opar et al., 2013; Giacomo et al., 2018; Lodge et al., 2020). They are innovative, reliable devices to measure eccentric hamstring strength, and have provided great insight into HSI prevention and muscle architecture alterations within recent research literature (Timmins et al., 2016; Giacomo et al., 2018; Presland et al., 2018; Hegyi et al., 2019; McGrath et al., 2020).

Some devices only measure linear force output and not torque about the knee, making measures unrelatable between players of different sizes. Moreover, they do not measure angular range over which the force can be produced. Such a relationship between joint angle and muscle length is important as previously injured muscles reach peak torque at significantly shorter lengths (40.9 ± 2.7°) than uninjured muscles of the opposite leg (29.8 ± 1.5° where 0° = full extension) (Brockett et al., 2004). A number of commercial devices have adapted the NHE by altering declination to minimize knee hyperextension or allow assistance to make the NHE easier to perform (Lodge et al., 2020). Other devices are assisted (Alt et al., 2018; Giacomo et al., 2018), which compromises the pure eccentric nature of the NHE, and removes the ability to achieve supramaximal intensity, which may limit its effectiveness as an injury prevention exercise. It should not be possible to control constant knee extension velocity and an accurate hip control throughout the entire range of motion (ROM) of the descent of a supramaximal eccentric NHE (Alt et al., 2020), as maximal hamstring torque production should lead to a loss of control and a subsequent break-point. Cuthbert et al. (2020b) states there is an argument for providing assistance to permit an increased ROM in weaker individuals or beginners, as long as a break-point occurs ensuring a supramaximal intensity. The aim of this study is to design a device to address some of the issues discussed and to manipulate the torque-length relationship of the knee flexors to elicit torque production at longer muscle lengths, mimicking the late swing phase injury location. A recent study by Šarabon et al. (2019) strengthens the hypothesis that performing the NHE on an incline will allow a participant to exercise at longer muscle lengths, whilst still being able to reach supramaximal torques. They reported that participants were able to reach similar peak knee and hip torques at longer estimated hamstring lengths using NHE slopes of 20 and 40°.

## Materials and Methods

### Experimental Design

There is little published literature available regarding the biomechanics of the NHE and an improved insight will assist in the interpretation of the relationship between the NHE and the torque-length relationship. Findings indicate that with consistent NHE training incorporating low repetitions, the angle of peak torque, and therefore break-point angle should increase (Cuthbert et al., 2020b). It is hypothesized that an increased break-point angle over time may be the intensity stimulus needed to maintain greater knee flexor adaptations (Cuthbert et al., 2020a), supporting the notion of it being a useful metric to monitor within HSI prevention. A custom-built dynamometer (HA*L*HAM°) was designed to manipulate the torque-angle relationship to elicit torque production at the longer muscle lengths, mimicking the late swing phase injury location. The HA*L*HAM° measures knee angle at the break-point and torque about the knee, making measures more relatable between players. Pilot work revealed “break-torque angle” (BTA) to be a key kinematic measure (representing the peak torque value and its corresponding relative thigh angle at that point) with the HA*L*HAM° using BTA to assess *proxy* muscle length changes i.e., the angular range over which the torque can be produced. To achieve this it uses adjustable inclinations, allowing an increase of NHE knee angle up to 20 degrees.

**Figure 1:**
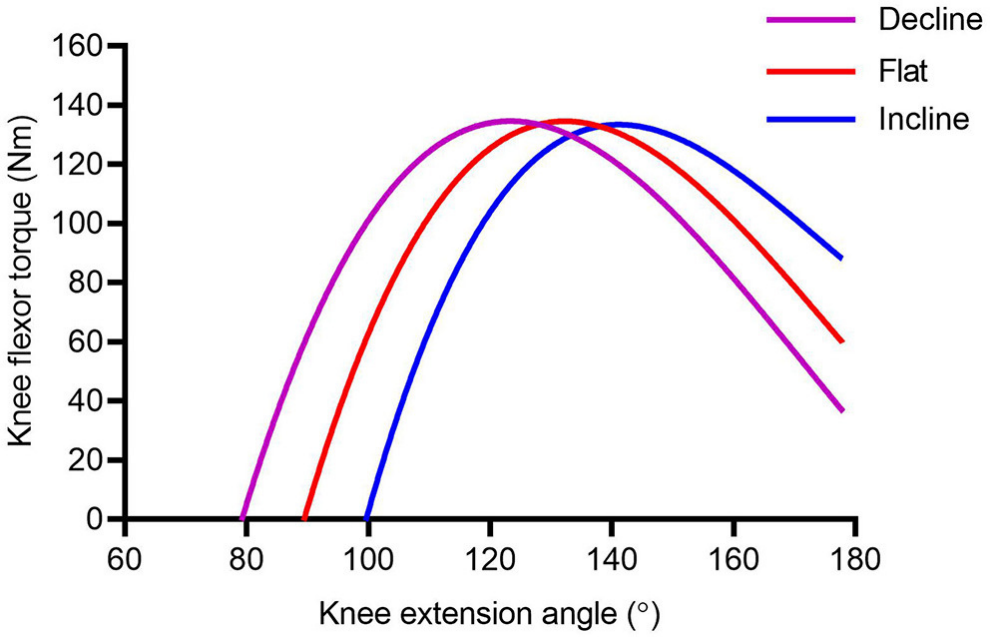
Schematic representation of the muscle torque-length relationship during the NHE performed at different inclinations. A rightward-shift in the torque-length relationship is hypothesized for the incline slope (blue line), and a leftward-shift hypothesized for the decline slope (purple line).

During the NHE a participant resists knee extension by producing increasing torque about the knee through eccentric action of the knee flexors. The torque increase is generated by the increasing moment arm around the knee, of a participant's center of mass, as they progress forwards. In a conventional NHE torque cannot be decoupled from muscle length however the same torque can be reached at different *proxy* muscle lengths by manipulating the shank angle. The angular mechanism targets the hamstrings capacity to apply torque over the more greatly extended knee angles, where injury is most likely to occur. During a conventional NHE the hamstrings are typically trained at short muscle lengths within 90–110° of knee extension range before the break-point is reached (Ditroilo et al., 2013). By altering the NHE relative knee angle using an incline slope, the same torques will be reached at a longer muscle length, resulting in a rightward-shift in the length-tension curve independent of overall hamstring strength (Figure 1). Conversely, when employing a decline position the same torques will be reached at shorter muscle lengths (Figure 1). Rightward-shifts in the torque-angle relationship could be of critical importance for athletes that require high eccentric hamstring force application at specific knee angles, such as those seen in high-speed running (Figure 1). Where the torque is being produced in the muscle is of importance as current research suggests having “strong and long” hamstring muscles is effectual for injury prevention (Bourne et al., 2015; Timmins et al., 2015) with lengthening hamstring exercises showing the fastest return-to-play and a lower reinjury rate compared with conventional hamstring exercises (Ishøi et al., 2020).

### Device and Metrics

**Figure 2:**
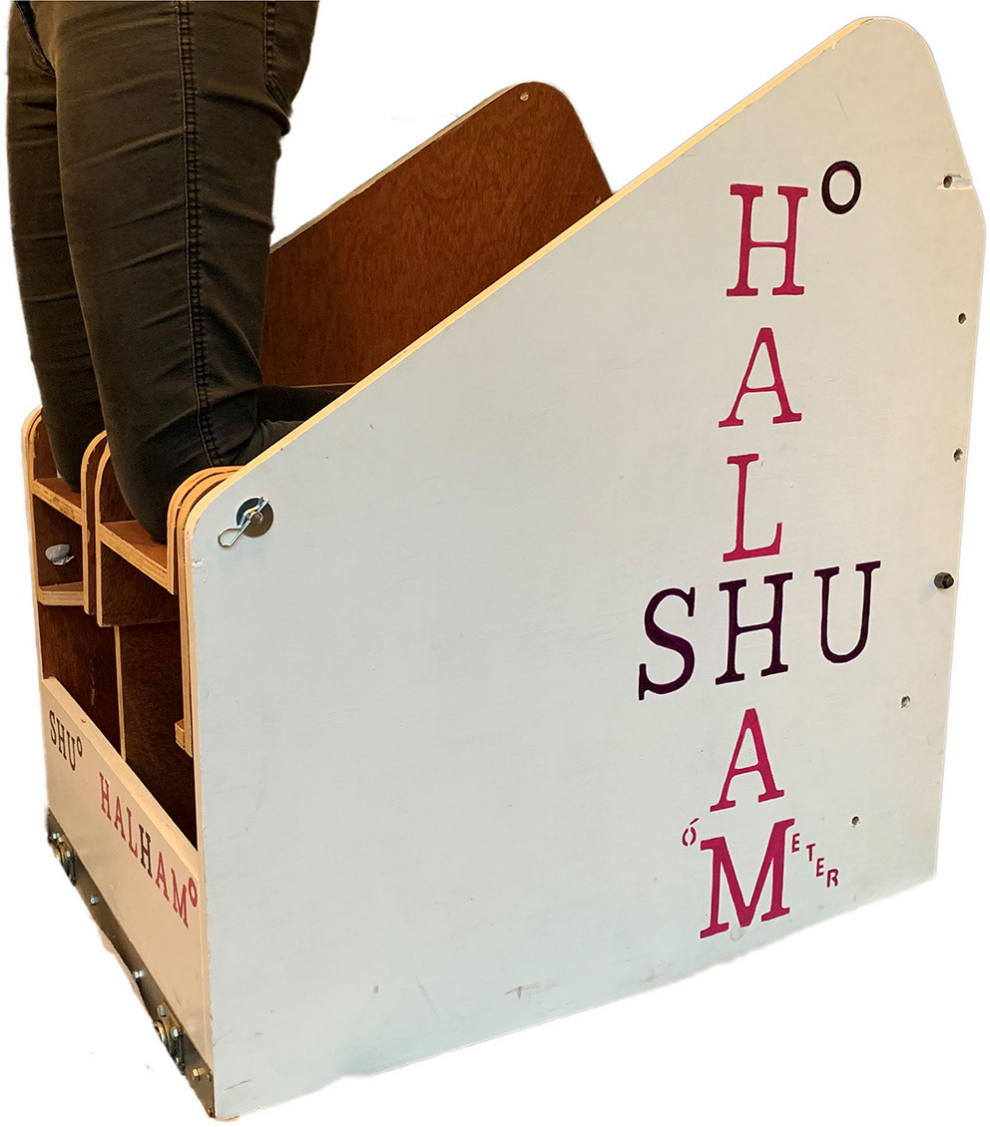
Prototype device image of the HA*L*HAM° showing participant positioning at the pivot point.

**Figure 3:**
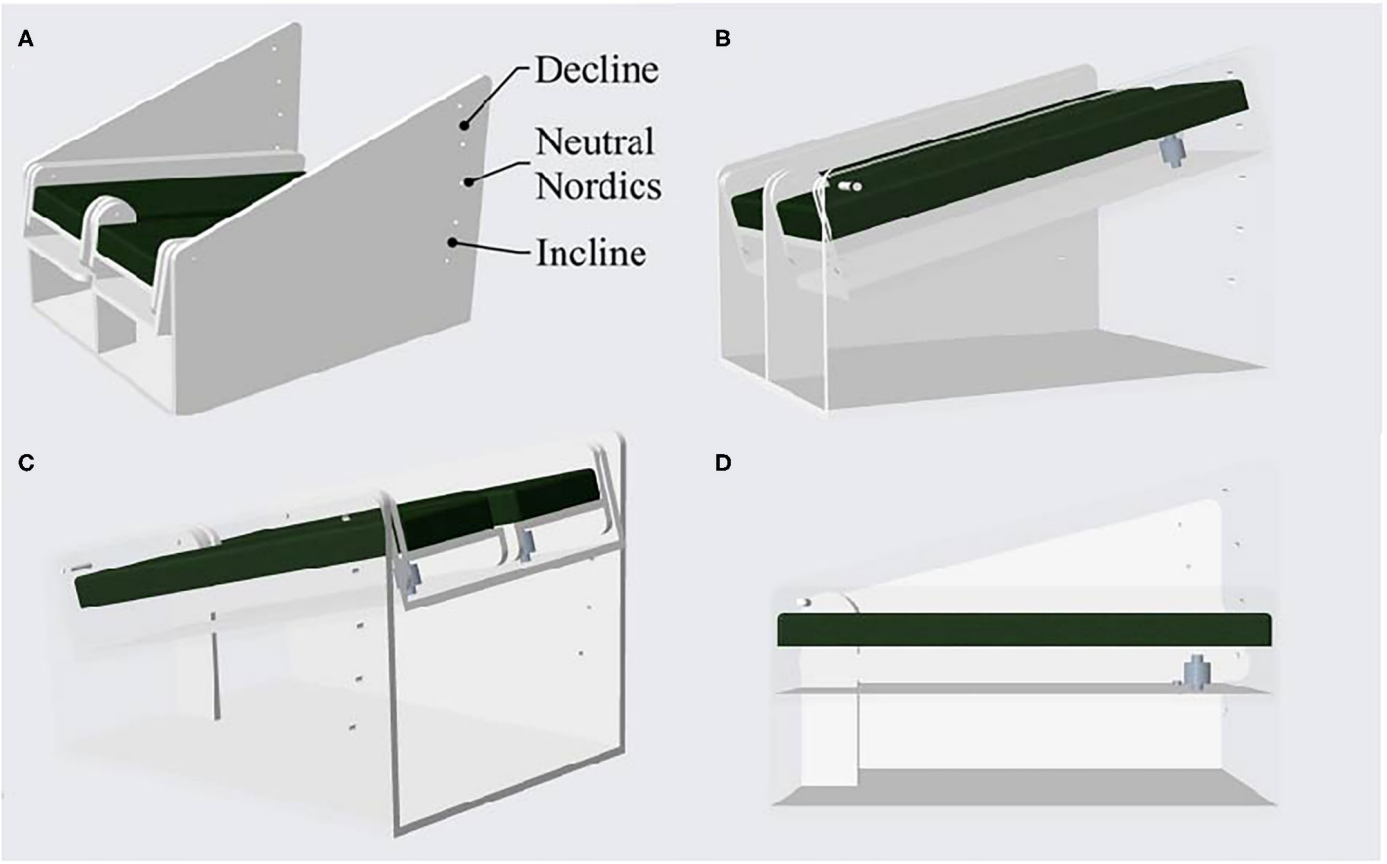
A computer-aided design of the HA*L*HAM° showing the prototype device **(A)**. The cut away side views show the positioning of the in-line strain gauge load cells and the moveable tray that is free to rotate **(B,D)**. Image **(C)** shows a cut-away rear view of the inclination mechanism over a range of plus and minus 20°.

Pilot work on a prototype device (HA*L*HAM°) explored the torque-angle relationships of the knee flexors (Figure 2). The HA*L*HAM° possesses a flat platform and the ankle straps are positioned 0.6 m from the pivot point at the front of the rig which determines the NHE start position, lining up the lateral femoral epicondyle of the femur with the pivot point before commencement. The novel rig includes a moveable mechanism allowing the shank incline to the horizontal to be altered at angle increments of 10° intervals (both incline and decline) over a range of plus and minus 20° (Figures 2, 3). The HA*L*HAM° uses strain gauge load cells (Omega, Engineering Inc. Norwalk, USA) attached at the rear in a fixed position within a moveable tray parallel to the shank, with the platform tray free to rotate (Figure 3). The load cells measure both individual right and left limb, and combined limb total forces producing force-time traces in line graph format, useful for determining individual leg strength and any bilateral force asymmetries during the NHE, as a >15% difference between limbs has been cited as a risk factor for HSI (Bourne et al., 2015). Torque is calculated for each NHE trial across all three inclinations from the force measured by the load cells and the distance measured from the set pivot point (0.6 m). Unilateral testing is possible on the HA*L*HAM° by securing only the testing limb into the ankle brace, however no unilateral NHE testing was performed due to the lack of familiarization of this exercise with the participants being tested. A laboratory grade 3-dimensional electromagnetic motion tracking system (Liberty® Polhemus, Colchester, Vermont, USA) was used to quantify angular displacement. The thigh sensor was positioned laterally on the upper leg equidistant from the greater trochanter and lateral femoral epicondyle. The trunk sensor was also positioned laterally equidistant from the greater trochanter and the shoulder bursa. Polhemus Liberty® software was used to collect kinematic data from the two sensors sampled at 240 Hz. Load cell data were sampled at 125 Hz via a Phidget Bridge data acquisition board (Phidgets Inc., Calgary, Canada) and exported in.CSV format to be processed on a personal computer. Further analysis was performed in spreadsheet software (Microsoft Excel software, Microsoft Corp., Redmond, Washington). Calculated parameters included:

- *Break-torque angle* (BTA) ~ representing the definitive bilateral peak eccentric knee flexor torque value and its corresponding thigh angle.
- *Peak torque* ~ NHE bilateral maximum torque value.
- *Relative trunk-to-thigh angle* (RTA) ~ the angle between the thigh and the trunk throughout the NHE ROM, relative to a fixed axis, representing hip angle. Subsequently, RTA at BTA angle was extracted from the data.
- *Angular velocity of the knee joint* (AVK) ~ representing the angular velocity of the knee joint throughout the NHE ROM, filtered using an 11-point average. Subsequently, AVK at BTA was extracted from the data.

### Trials

Eighteen recreationally active male rugby union players (*n* = 18) of varying playing experience were recruited to participate in this study (mean ± SD age 20 ± 3years, height 182 ± 6.7 cm, and body mass 91.0 ± 47.4 kg). Participants completed a questionnaire, used to gather injury data and personal characteristics. All participants reported having some previous training history of performing the NHE. Two participants reported a previous Grade 1 thigh muscle injury in the last 6 months but were stated as being physically fit and not currently carrying an injury which would affect them completing the NHE trials. All other participants had no lower-limb injuries reported in the last 12 months. Exclusion criterion included any participants not medically cleared from disease or any person carrying an injury that would affect performance of the NHE. Participants were recruited from the same sport and team level to ensure gathered data would be based on players with similar conditioning levels. Females were excluded to prevent physiological differences between genders and because male soccer players are 64% more likely to sustain an HSI, and suffer a higher proportion of recurrent HSIs compared with females (men 22% vs. women 12%) (Cross et al., 2013). After having all procedures explained to them, participants provided written informed consent to participate in the spirit of the Helsinki Declaration, before testing commenced. Ethical approval for the study was approved by the Sheffield Hallam University Ethics Committee. Prior to performing the trials participants were instructed to perform a standardized warm-up by using a stationary bike for 3 min and completing dynamic movements such as leg swings, walking lunges, and squats (two sets of 10 repetitions). Participants assumed a starting 90° kneeling position on the HA*L*HAM° device with their hips fully extended and their ankles were secured in place. Participants were asked to tap the thigh sensor at the beginning of each NHE trial and then gradually lean forward at the slowest possible speed, keeping their hips fixed in line with their knee and shoulder joints throughout the ROM. They were instructed to keep their trunk in a neutral position throughout, with hands facing forward and elbows pointing down, ready to buffer the fall. This action was performed until the participant could no longer withstand the torque around their knees caused by the increasing moment arm of their mass as they leaned forwards (Petersen et al., 2011; Sconce et al., 2015). A warm-up set of three submaximal bilateral NHEs were performed prior to one set of three maximal bilateral NHEs at a standard 0° flat angle position (FLAT), a 10° incline (INC), and a 10° decline angle (DEC). Nine trials per person were collected with each participant performing a set of 3 reps at one inclination before resting for 2–5 min until all three inclinations had been performed. The inclination order was randomized between participants. The rest period between reps was long enough to allow the participant to comfortably re-set themselves for the next maximal effort. Verbal encouragement was given by researchers throughout the testing to ensure maximal effort.

162 trials from 18 participants were initially considered (54 trials for each of the three angles used). Ten mistrials or mis-recordings were discounted, including the absence of a tap signal on the thigh sensor as this affected the synchronization process, consequentially leaving 152 trials. Further to this, one participant who reached full extension during all nine trials was removed. From the remaining 143 trials the average peak torque for each trial for both limbs (left, right, and combined) and BTA were calculated. Bourne et al. (2019) states that a NHE trial is deemed acceptable when the force output reaches a distinct peak (indicative of maximal eccentric strength), followed by a rapid decline in force, occurring when the participant can no longer resist the effects of gravity. By graphically analyzing data in Excel, trials were rejected when there was no clear peak, an extended flattened period, or when there was no clear torque drop-off period, leaving 127 trials remaining for analysis. The subsequent angle reached at break-torque was calculated from the accumulated increasing angle during the descent toward full knee extension angle (180°).

### Statistical Analysis

The data were statistically processed in GraphPad Prism 8.43 (GraphPad Software Inc). Descriptive statistics for all the remaining 127 trials were calculated and reported as mean ± standard deviation (Table 1). The D'Agostino-Pearson test was used for testing of normality. One-way repeated measures ANOVA were performed to identify differences between eccentric knee flexor torque (Nm), AVK (deg·s^−1^), and RTA (°), at BTA (°) in different inclinations (Table 1). Where significant effects were detected, *post-hoc t*-tests with Tukey's HSD were applied to determine where any mean differences occurred. The level of statistical significance was set at *p* <0.05 for all analyses. Mean differences of all measurements were reported with their 95% confidence intervals. Where appropriate, effects sizes were calculated by Hedges' g and interpreted as small (g > 0.2), moderate (g > 0.5), and large (g > 0.8).

**Table 1:** Mean ± SD for each variable considered in the study.

**Variables**	**Mean±SD**	**Range (Min-Max)**
**Torque (Nm)**		
Decline	132.0 ± 63.1	20.1–246.1
Flat	149.7 ± 70.1	50.5–316.7
Incline	148.9 ± 64.9	72.3–275.4
**Break-torque angle (°)**		
Decline	112.1 ± 8.3*^a^^c^*	98.5–131.7
Flat	126.0 ± 9.8*^a^^b^*	108.8–149.4
Incline	134.1 ± 8.6*^b^^c^*	118.8–151.6
**Angular velocity of the knee joint at peak torque (deg·s**^**−1**^**)**		
Decline	23.8 ± 14.4	7.3–68.2
Flat	29.2 ± 22.6	3.6–93.4
Incline	24.5 ± 22.6	4.5–96.3
**Relative thigh-to-trunk angle at peak torque (°)**		
Decline	20.4 ± 10.4	1.8–53.8
Flat	16.7 ± 10.8	0.4–44.7
Incline	20.2 ± 11.2	3.1–51.1

*Variability (range) stated for relevant metrics. The effect of different inclinations on eccentric knee flexor torque and break-torque angle shown*.

a *Denotes significant difference between Decline vs. Flat (p <0.01)*.

b *Denotes significant difference between Flat vs. Incline (p <0.01)*.

c *Denotes significant difference between Decline vs. Incline (p <0.01)*.

## Results

### Peak Torque and Break-Torque Angle

**Figure 4:**
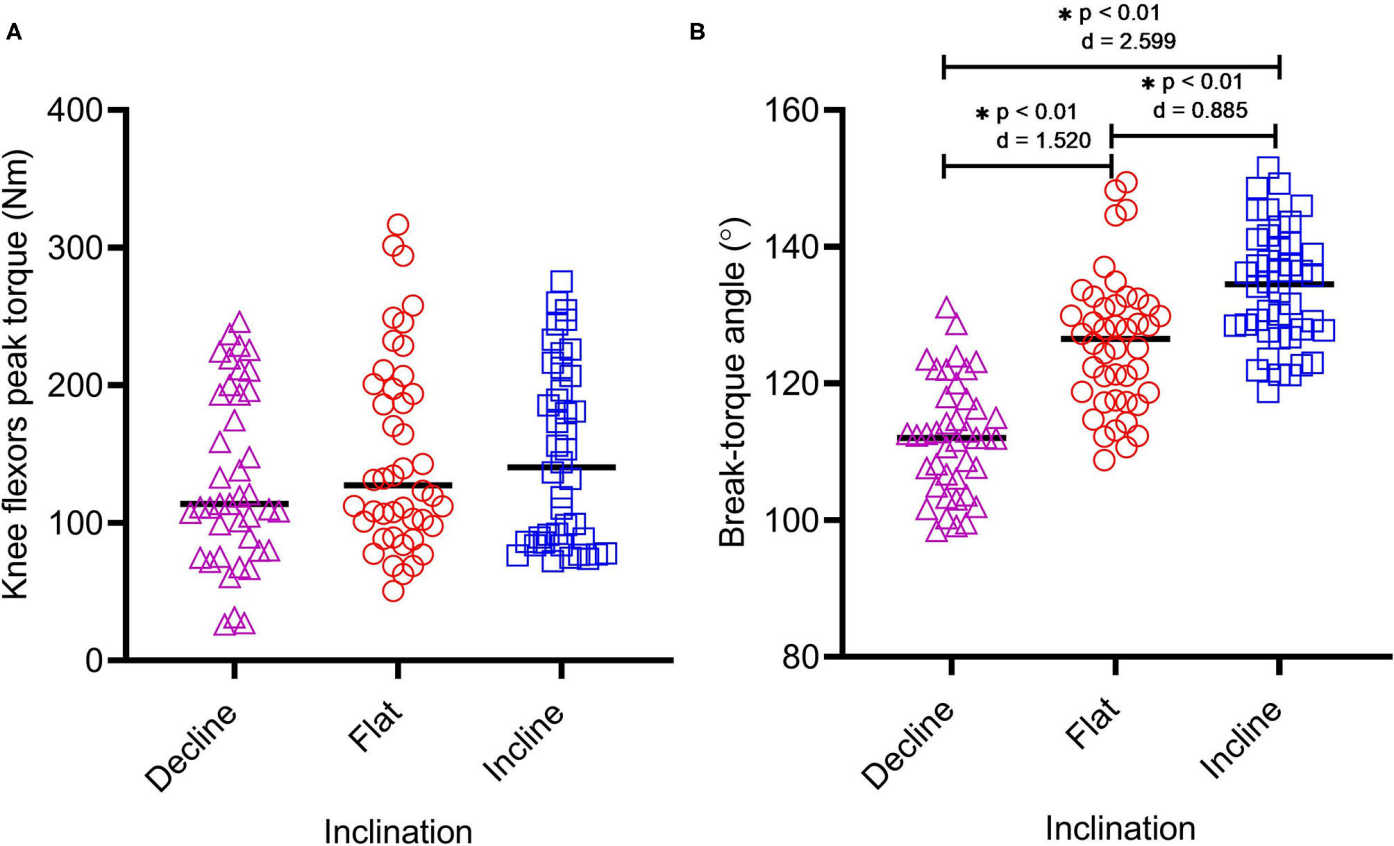
Eccentric knee flexor peak torque **(A)** and break-torque angle **(B)** at each inclination (DEC *n* = 41, FLAT *n* = 44, and INC *n* = 42) whilst performing the Nordic hamstring exercise on the HA*L*HAM°. Asterisks (*) indicate any significant differences between inclination.

Altering inclination did not affect eccentric knee flexor peak torque (*F* = 0.952, *p* = 0.389). Peak torques at altered inclinations can be seen in Table 1 and Figure 4. Changing inclination significantly affected BTA (*F* = 63.85, *p* <0.01), which increased when the NHE was performed at INC (134.1 ± 8.6°) compared to both the DEC (112.1 ± 8.3°) (*p* <0.01, *g* = 2.599) and conventional FLAT NHE (126.0 ± 9.8°) (*p* <0.01, *g* = 0.885) (Table 1). Changes in mean BTAs at altered inclinations can be seen in Table 1 and Figure 4.

### Angular Velocity of the Knee Joint, and Relative Trunk-to-Thigh Angle at Break-Torque

**Figure 5:**
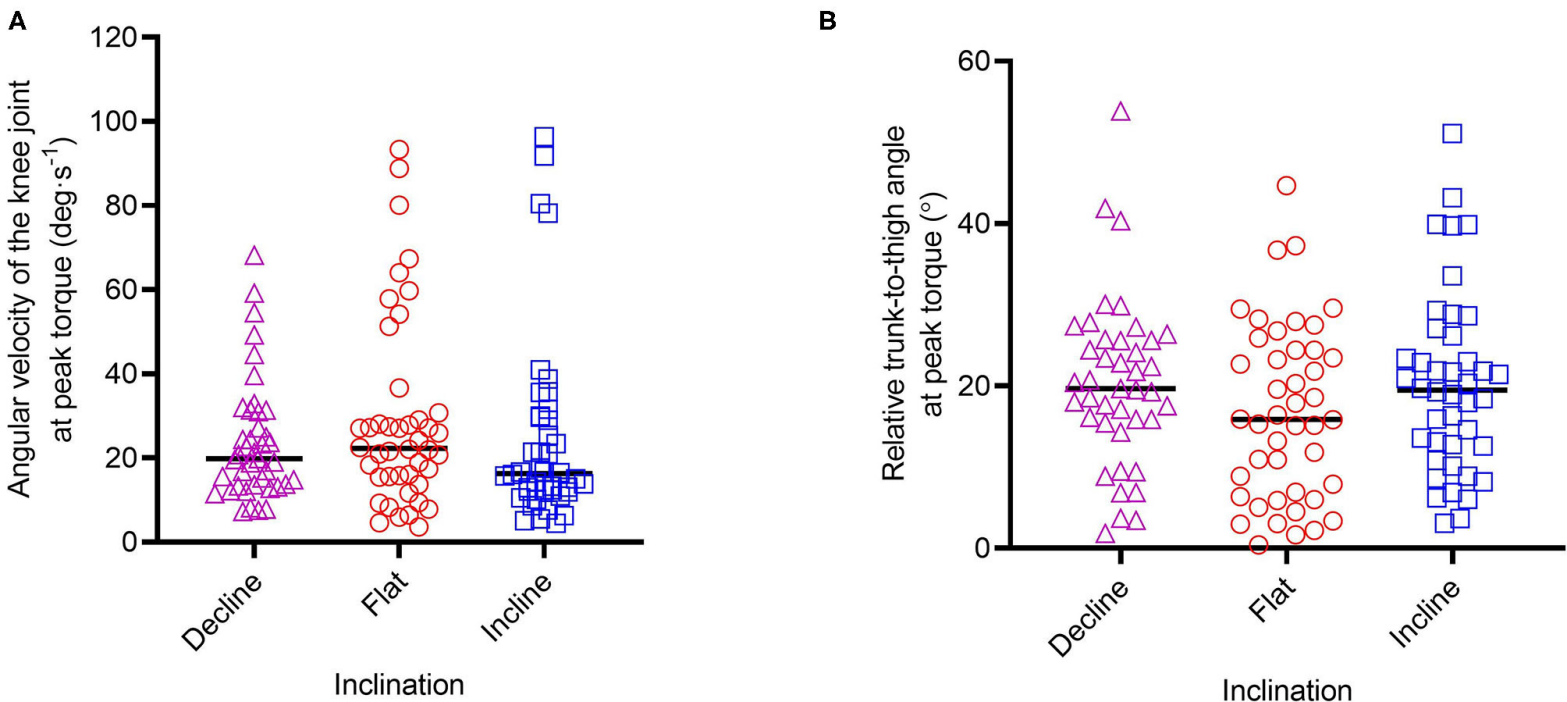
Angular velocity of the knee joint **(A)** and relative trunk-to-thigh angle **(B)**, at peak torque across the inclinations.

Altering inclination had no significant effect on the angular velocity of the knee joint (AVK) (*F* = 0.880, *p* = 0.418) however large variability was reported for AVK at BTA in all inclinations, with FLAT reporting the highest mean difference (29.2 ± 22.6 deg·s^−1^). Altering inclination had no significant effect on RTA (*F* = 1.597, *p* = 0.207) however large variability can be seen for RTA at BTA in all inclinations, with DEC reporting the highest mean difference and range between trials (20.4 ± 10.4°; 1.8–53.8°) (Table 1). AVK and RTA at different inclinations can be seen in Figure 5.

## Discussion

The findings demonstrate the hypothesized rightward shift (Figure 1) of (*proxy)* muscle length. NHE BTA significantly increased when performed at INC compared to both the DEC (*p* <0.01, *g* = 2.599) and standard NHE (*p* = 0.0002, *g* = 0.885) (Table 1) however, there was no significant change in peak torque across the 3 inclinations (*p* = 0.389). This suggests that an incline NHE may be able to train the knee flexor muscles at more extended lengths, similar to those seen in sprinting where the site of most HSIs occur. This rationale has been strengthened by recent research conducted by Šarabon et al. (2019), where a custom device was used to alter the slope of the starting knee angle when performing a NHE. They reported that modified variations of the NHE (20 and 40° slopes) allowed participants to reach peak knee and hip torques at longer estimated hamstring lengths (20° slope = 75.0 ± 7.3°; 40° slope = 87.9 ± 7.5°) compared to the conventional flat NHE (56.1 ± 9.1°). Using the HA*L*HAM° incline slope for eccentric long-length muscle training may favorably affect architectural adaptations within the knee flexor muscles, such as increased fascicle length and muscle hypertrophy. However, further studies are needed to confirm the occurrence of these adaptations and if they are greater when compared to using the conventional (FLAT) NHE. Relative specific strength, where an individual may possess a comparatively greater eccentric strength at a particular slope angle, should also be considered in terms of its relationship to NHE performance and HSI prevention.

It should be noted that ankle position on the HA*L*HAM° is self-determined by the participant i.e., plantar-flexed or dorsi-flexed, which is the same method traditionally employed during a floor NHE. One limitation of this approach could be that the ankle isn't fixed in a particular position, however it has been reported that ankle position does not influence biceps femoris normalized muscle activity during the NHE (dorsi-flexed = 124.5 ± 6.2% vs. plantar-flexed = 128.1 ± 5.0%, *p* > 0.05, Cohen d = 0.64) (Comfort et al., 2017), which is important as HSIs primarily affect the biceps femoris long head muscle in sprinting sports (Askling et al., 2007). It is well-recognized that a multifactorial approach should be considered in HSI prevention. Any device focused on specific individual risk factors should therefore only be considered as an additional tool to inform injury data (associations rather than prediction) and return-to-play outcomes. Limitations of the current study include a lack of NHE familiarization and inter-individual variability in technique. Although participants were instructed to remain fully extended at the hip throughout the movement and perform a slow, controlled descent until supramaximal failure, this was difficult to enforce and control between trials. As a result, a wide range of hip positions and knee joint angular velocity at BTA were recorded at each inclination. However, altering the inclination had no significant effect on AVK (*F* = 0.880, *p* = 0.418) or RTA (*F* = 1.597, *p* = 0.207) (Table 1; Figure 5) and it can be assumed therefore that differing inter-individual technique rather than inclination was the cause of these effects. Future work will look to test a large pool of participants that have undergone NHE familiarization before testing. Future directives will be to integrate a feedback system to better control hip position and knee extension speed, and thus explore the influence of technique on angular-torque metrics.

### Conclusion

The HA*L*HAM° allows modification and measurement of the torque-angle relationship of the knee flexors and can target the knee flexors at longer lengths using an incline slope mechanism. Monitoring the interplay of the torque-length relationship trade-off over a period of time, i.e., where in the muscle peak torque is being produced (“*strong* and *long*” analogy of the knee flexors), may help determine those at greater risk of sustaining a HSI and aid a gradual return-to-play.

## Data Availability Statement

The raw data supporting the conclusions of this article will be made available by the authors, without undue reservation.

## Ethics Statement

The studies involving human participants were reviewed and approved by Sheffield Hallam University Research Ethics Committee. The patients/participants provided their written informed consent to participate in this study.

## Author Contributions

All authors (ES, BH, TM-W, and NH) contributed to the study conception and design. Material preparation and method protocols had contributions from all authors. ES was involved in the data collection. All authors contributed to the article and approved the submitted version.

## Conflict of Interest

The authors declare that the research was conducted in the absence of any commercial or financial relationships that could be construed as a potential conflict of interest.

## Abbreviations

AVK: Angular velocity of the knee joint
BTA: Break-torque angle
DEC: Decline slope
FLAT: Flat position
HSI: Hamstring strain injury
INC: Incline slope
NHE: Nordic hamstring exercise
ROM: Range of motion
RTA: Relative trunk-to-thigh angle.

## References

[B1] Alt T. Nodler Y. T. Severin J. Knicker A. J. Strüder H. K. (2018). Velocity specific and time-dependent adaptations following a standardized Nordic hamstring exercise training. Scand. J. Med. Sci. Sports 28, 65–76. 10.1111/sms.12868 28247444

[B2] Alt T. Nodler Y. T. Severin J. Knicker A. J. Strüder H. K. (2020). Comment on: “The effect of Nordic hamstring exercise intervention volume on eccentric strength and muscle architecture adaptations: a systematic review and meta-analyses.” Sports Med. 50, 219–221. 10.1007/s40279-019-01244-0 31820377

[B3] Arnason A. Andersen T. E. Holme I. Engebretsen L. Bahr R. (2008). Prevention of hamstring strains in elite soccer: an intervention study. Scand. J. Med. Sci. Sports 18, 40–48. 10.1111/j.1600-0838.2006.00634.x 17355322

[B4] Askling C. M. Tengvar M. Saartok T. Thorstensson A. (2007). Acute first-time hamstring strains during high-speed running: a longitudinal study including clinical and magnetic resonance imaging findings. Am. J. Sports Med. 35, 197–206. 10.1177/0363546506294679 17170160

[B5] Bourne M. N. Bruder A. M. Mentiplay B. F. Carey D. L. Patterson B. E. Crossley K. M. (2019). Eccentric knee flexor weakness in elite female footballers 1–10 years following anterior cruciate ligament reconstruction. Phys. Ther. Sport 37, 144–149. 10.1016/j.ptsp.2019.03.010 30959444

[B6] Bourne M. N. Opar D. A. Williams M. D. Shield A. J. (2015). Eccentric knee flexor strength and risk of hamstring injuries in rugby union. Am. J. Sports Med. 43, 2663–2670. 10.1177/0363546515599633 26337245

[B7] Brockett C. L. Morgan D. L. Proske U. (2004). Predicting hamstring injury in elite athletes. Med. Sci. Sports Exerc. 36, 379–387. 10.1249/01.MSS.0000117165.75832.05 15076778

[B8] Brockett C. L. Morgan D. L. Proske U. W. E. (2001). Human hamstring muscles adapt to eccentric exercise by changing optimum length. Med. Sci. Sports Exerc. 33, 783–790. 10.1097/00005768-200105000-00017 11323549

[B9] Brughelli M. Cronin J. (2007). Altering the length-tension relationship with eccentric exercise: implications for performance and injury. Sports Med. 37, 807–826. 10.2165/00007256-200737090-00004 17722950

[B10] Comfort P. Regan A. Herrington L. Thomas C. McMahon J. Jones P. (2017). Lack of effect of ankle position during the Nordic curl on muscle activity of the biceps femoris and medial gastrocnemius. J. Sport Rehabil. 26, 202–207. 10.1123/jsr.2015-0130 27632836

[B11] Cross K. M. Gurka K. K. Saliba S. Conaway M. Hertel J. (2013). Comparison of hamstring strain injury rates between male and female intercollegiate soccer athletes. Am. J. Sports Med. 41, 742–748. 10.1177/0363546513475342 23408592

[B12] Cuthbert M. Ripley N. Mcmahon J. J. Evans M. Haff G. G. Comfort P. (2020a). Reply to: “Comment on: the effect of Nordic hamstring exercise intervention volume on eccentric strength and muscle architecture adaptations: a systematic review and meta-analyses.” Sports Med. 50, 223–225. 10.1007/s40279-019-01245-z 31820375

[B13] Cuthbert M. Ripley N. Mcmahon J. J. Evans M. Haff G. G. Comfort P. (2020b). The effect of Nordic hamstring exercise intervention volume on eccentric strength and muscle architecture adaptations: a systematic review and meta-analyses. Sports Med. 50, 83–99. 10.1007/s40279-019-01178-7 31502142 PMC6942028

[B14] Ditroilo M. De Vito G. Delahunt E. (2013). Kinematic and electromyographic analysis of the Nordic Hamstring Exercise. J. Electromyogr. Kinesiol. 23, 1111–1118. 10.1016/j.jelekin.2013.05.008 23809430

[B15] Ekstrand J. Hägglund M. Waldén M. (2011). Epidemiology of muscle injuries in professional football (soccer). Am. J. Sports Med. 39, 1226–1232. 10.1177/0363546510395879 21335353

[B16] Ekstrand J. Waldén M. Hägglund M. (2016). Hamstring injuries have increased by 4% annually in men's professional football, since 2001: a 13-year longitudinal analysis of the UEFA Elite Club injury study. Br. J. Sports Med. 50, 731–737. 10.1136/bjsports-2015-095359 26746908

[B17] Franchi M. V. Reeves N. D. Narici M. V. Huey K. (2017). Skeletal muscle remodeling in response to eccentric vs. concentric loading: morphological, molecular, and metabolic adaptations. Front. Physiol. 8:447. 10.3389/fphys.2017.00447 28725197 PMC5495834

[B18] Giacomo J. Lahti J. Hegyi A. Gerus P. Morin J. (2018). A new testing and training device for hamstring muscle function. Sport Perform. Sci. Rep. 40, 1–4. Available online at: https://sportperfsci.com/a-new-testing-and-training-device-for-hamstring-muscle-function/

[B19] Green B. Bourne M. N. Pizzari T. (2018). Isokinetic strength assessment offers limited predictive validity for detecting risk of future hamstring strain in sport: a systematic review and meta-analysis. Br. J. Sports Med. 52, 329–336. 10.1136/bjsports-2017-098101 29187349

[B20] Guex K. Gojanovic B. Millet G. P. (2012). Influence of hip-flexion angle on hamstrings isokinetic activity in sprinters. J. Athl. Train. 47, 390–395. 10.4085/1062-6050-47.4.04 22889654 PMC3396298

[B21] Hägglund M. Waldén M. Ekstrand J. (2006). Previous injury as a risk factor for injury in elite football: a prospective study over two consecutive seasons. Br. J. Sports Med. 40, 767–772. 10.1136/bjsm.2006.026609 16855067 PMC2564391

[B22] Hegyi A. Lahti J. Giacomo J.-P. Gerus P. Cronin N. J. Morin J.-B. (2019). Impact of hip flexion angle on unilateral and bilateral Nordic hamstring exercise torque and high-density electromyography activity. J. Orthop. Sports Phys. Ther. 49, 584–592. 10.2519/jospt.2019.8801 30913969

[B23] Ishøi L. Krommes K. Husted R. S. Juhl C. B. Thorborg K. (2020). Diagnosis, prevention, and treatment of common lower extremity muscle injuries in sport—grading the evidence: a statement paper commissioned by the Danish Society of Sports Physical Therapy (DSSF). Br. J. Sports Med. 54, 528–537. 10.1136/bjsports-2019-101228 31937579 PMC7212929

[B24] Lodge C. Tobin D. Rourke B. O. Thorborg K. (2020). Reliability and validity of a new eccentric hamstring strength measurement device. Arch. Rehabil. Res. Clin. Transl. 2, 1–6. 10.1016/j.arrct.2019.100034 33543063 PMC7853328

[B25] McGrath T. M. Hulin B. T. Pickworth N. Clarke A. Timmins R. G. (2020). Determinants of hamstring fascicle length in professional rugby league athletes. J. Sci. Med. Sport 23, 524–528. 10.1016/j.jsams.2019.12.006 31928881

[B26] Morgan D. L. (1990). New insights into the behavior of muscle during active lengthening. Biophys. J. 57, 209–221. 10.1016/S0006-3495(90)82524-8 2317547 PMC1280663

[B27] Opar D. A. Williams M. Piatkowski T. Shield A. (2013). A novel field test of eccentric hamstring strength: a reliability and injury study. J. Sci. Med. Sport 16:e41. 10.1016/j.jsams.2013.10.098

[B28] Opar D. A. Williams M. D. Shield A. J. (2012). Hamstring strain injuries: factors that lead to injury and re-Injury. Sports Med. 42, 209–226. 10.2165/11594800-000000000-00000 22239734

[B29] Oranchuk D. J. Storey A. G. Nelson A. R. Cronin J. B. (2019). Isometric training and long–term adaptations: effects of muscle length, intensity, and intent: a systematic review. Scand. J. Med. Sci. Sports 29, 484–503. 10.1111/sms.13375 30580468

[B30] Petersen J. Thorborg K. Nielsen M. B. Budtz-Jørgensen E. Hölmich P. (2011). Preventive effect of eccentric training on acute hamstring injuries in men's soccer: a cluster-randomized controlled trial. Am. J. Sports Med. 39, 2296–2303. 10.1177/0363546511419277 21825112

[B31] Potier T. G. Alexander C. M. Seynnes O. R. (2009). Effects of eccentric strength training on biceps femoris muscle architecture and knee joint range of movement. Eur. J. Appl. Physiol. 105, 939–944. 10.1007/s00421-008-0980-7 19271232

[B32] Presland J. Timmins R. Bourne M. Williams M. Opar D. (2018). The effect of high or low volume Nordic hamstring exercise training on eccentric strength and biceps femoris long head architectural adaptations. J. Sci. Med. Sport 28, 1775–1783. 10.1016/j.jsams.2017.09.213 29572976

[B33] Šarabon N. Marušič J. Marković G. Kozinc Ž. (2019). Kinematic and electromyographic analysis of variations in Nordic hamstring exercise. PLoS ONE 14:e0223437. 10.1371/journal.pone.0223437 31644582 PMC6808554

[B34] Sconce E. Jones P. Turner E. Comfort P. Graham-Smith P. (2015). The validity of the Nordic hamstring lower for a field-based assessment of eccentric hamstring strength. J. Sport Rehabil. 24, 13–20. 10.1123/jsr.2013-0097 25606859

[B35] Timmins R. G. Bourne M. N. Shield A. J. Williams M. D. Lorenzen C. Opar D. A. (2016). Short biceps femoris fascicles and eccentric knee flexor weakness increase the risk of hamstring injury in elite football (soccer): a prospective cohort study. Br. J. Sports Med. 50, 1524–1535. 10.1136/bjsports-2015-095362 26675089

[B36] Timmins R. G. Shield A. J. Williams M. D. Lorenzen C. Opar D. A. (2015). Biceps femoris long head architecture: a reliability and retrospective injury study. Med. Sci. Sports Exerc. 47, 905–913. 10.1249/MSS.0000000000000507 25207929

[B37] Van Der Horst N. Smits D. W. Petersen J. Goedhart E. A. Backx F. J. G. (2015). The preventive effect of the Nordic hamstring exercise on hamstring injuries in amateur soccer players: a randomized controlled trial. Am. J. Sports Med. 43, 1316–1323. 10.1177/0363546515574057 25794868

[B38] Van Dyk N. Bahr R. Burnett A. F. Whiteley R. Bakken A. Mosler A.. (2017). A comprehensive strength testing protocol offers no clinical value in predicting risk of hamstring injury: a prospective cohort study of 413 professional football players. Br. J. Sports Med. 51, 1695–1702. 10.1136/bjsports-2017-097754 28756392

[B39] Van Dyk N. Behan F. P. Whiteley R. (2019). Including the Nordic hamstring exercise in injury prevention programmes halves the rate of hamstring injuries: a systematic review and meta-analysis of 8459 athletes. Br. J. Sports Med. 53, 1362–1370. 10.1136/bjsports-2018-100045 30808663

[B40] Van Hooren B. Bosch F. (2017). Is there really an eccentric action of the hamstrings during the swing phase of high-speed running? Part II: implications for exercise. J. Sports Sci. 35, 2322–2333. 10.1080/02640414.2016.1266019 27935419

